# Intergenic Variants Upstream of GADD45b Affect Survival of *Micropterus salmoides* Following LMBV Exposure

**DOI:** 10.3390/ijms26199281

**Published:** 2025-09-23

**Authors:** Pinhong Li, Xia Luo, Wenxian Li, Xiaozhe Fu, Qiang Lin, Yinjie Niu, Hongru Liang, Baofu Ma, Wenwen Xiao, Ningqiu Li

**Affiliations:** 1 College of Fisheries and Life Science, Shanghai Ocean University, Shanghai 201306, China; d220100042@st.shou.edu.cn (P.L.); lwx1369782725@163.com (W.L.); 2 Pearl River Fisheries Research Institute, Chinese Academy of Fishery Sciences, Key Laboratory of Fishery Drug Development, Ministry of Agriculture and Rural Affairs, Guangdong Province Key Laboratory of Aquatic Animal Immune and Sustainable Aquaculture, Guangzhou 510380, China; lxwenhao@163.com (X.L.); fuxiaozhe-1998@163.com (X.F.); lin9902057@163.com (Q.L.); niuyinjie0530@163.com (Y.N.); hrliang13@126.com (H.L.); mabf@prfri.ac.cn (B.M.); xiaowenwen217@163.com (W.X.)

**Keywords:** *Micropterus salmoides*, largemouth bass ranavirus (LMBV), growth arrest and DNA damage 45b (GADD45b), intergenic polymorphisms

## Abstract

GADD45 (growth arrest and DNA damage inducible 45) is a crucial signaling regulator in cells and plays an important role in various biological processes, including cellular stress response, cell cycle control, DNA damage repair, apoptosis, and tumor suppression. Our previous studies identified GADD45b as a candidate gene associated with resistance to largemouth bass ranavirus (LMBV) infection in largemouth bass (*Micropterus salmoides*). In the present study, the upstream intergenic polymorphisms of GADD45b were investigated to explore their association with resistance/susceptibility to LMBV. We employed the kompetitive allele specific PCR (KASP) assay to genotype 118 resistant individuals and 122 susceptible individuals following LMBV infection. The results revealed that SNP38943374 C>A and SNP38943495 G>A were significantly associated with LMBV resistance/susceptibility (*p* < 0.01). Individuals with the CC genotype of SNP38943374 and the GG genotype of SNP38943495 were more prevalent in resistant groups and have advantages in survival time after LMBV infection. Linkage disequilibrium analysis indicated strong linkage among these two loci. The distinct dynamic expression patterns of GADD45b in different genotypes following LMBV infection suggest its functional role in viral infection. Additionally, dynamic expression levels of immune-related genes (IFN-γ, TNF-α, and IL-10) also varied among different genotypes. These results demonstrated that the two SNPs in GADD45b could be used as candidate markers for further investigation of selective breeding of resistant largemouth bass to LMBV.

## 1. Introduction

Aquatic animals are highly vulnerable to pathogens under farming conditions, and disease outbreaks often result in significant economic losses. Consequently, genetic improvement of disease resistance has become a major focus in aquaculture breeding programs. As a molecular marker-assisted breeding method, single nucleotide polymorphisms (SNPs) have been extensively utilized in aquaculture breeding [1]. In genetic studies of aquatic species, researchers have identified a large number of SNP loci closely associated with economically important traits, including stress resistance [2,3,4], growth traits [5,6,7,8], sex-related traits [9,10] and disease resistance [11,12,13].

The growth arrest and DNA damage inducible 45 (GADD45) family, including GADD45a, GADD45b, and GADD45g, is extensively involved in diverse biological pathways including cell cycle regulation, DNA repair, apoptosis, cellular stress response, and immune regulation [14,15]. GADD45b, also known as MyD118, contains conserved DNA elements including c-Rel, USF/N-myc, NF-Y, GC box, CCAAT box and TATA box [16]. Previous studies have reported the expression of GADD45b was modulated by the immune-related transcription factors NF-κB and p53 [16]. It has been revealed that GADD45b plays an important role in the process of viral infection. Human immunodeficiency virus (HIV)-1 and avian leukosis virus subgroup J (ALV-J) infection induced the expression of GADD45b, which contributed to apoptosis and autophagy [17,18,19]. Furthermore, overexpression of GADD45b was found to specifically inhibit HIV-1 promoter activity, whereas knockdown of GADD45b could activate the latent of HIV-1 [18]. On the other hand, GADD45b influences various biological processes by regulating immune-related signaling pathways. Studies have demonstrated that GADD45b regulates autophagy, apoptosis and cell differentiation through the p38 MAPK signaling pathway [20,21,22]. GADD45b regulated the chemotactic response of granulocytes and macrophages to LPS via the p38/JNK signaling pathway [23,24]. Previous studies have demonstrated that GADD45b mediated the activation of MAPK signaling in Th1 cells, thereby influencing interferon-gamma (IFN-γ) production [25]. Thus, Gadd45b functions as an essential feedback regulator that sustains both cognate and inflammatory signaling [25]. Given the critical role of GADD45b in viral infections and host immune responses, it is worth investigating the association between GADD45b gene polymorphisms and disease resistance.

The largemouth bass (*Micropterus salmoides*) is an economically important freshwater fish species in China. In intensive aquaculture systems, largemouth bass are frequently suffered by various diseases caused by viral, bacterial, and parasitic pathogens, resulting in substantial economic losses to the farming industry. Largemouth bass virus (LMBV) is a prevalent viral pathogen in largemouth bass aquaculture [26,27]. Infected fish typically present clinical signs including skin and muscle ulceration, as well as splenic and hepatic swelling [26]. To date, preventive and control measures against LMBV-induced diseases have primarily focused on vaccine development and drugs applications [28,29]. However, challenges persist due to antigenic variability in field LMBV strains and drug resistance development. This has stimulated the adoption of molecular marker-assisted disease-resistant breeding as an alternative solution.

In previous studies, we identified GADD45b as a candidate gene associated with LMBV resistance in largemouth bass through genome-wide association studies (GWAS) [30]. In the present study, SNPs of GADD45b were investigated to explore their association with resistance/susceptibility to LMBV. We compared the survival times of individuals of different genotypes after LMBV infection. We also investigated the dynamic expression patterns of GADD45b and immune-related genes in individuals of different genotypes after viral infection. These results could provide potential molecular markers for the breeding of largemouth bass resistant to LMBV.

## 2. Results

### 2.1. Polymorphisms in GADD45b Gene and Their KASP-Based Genotyping Profiles

**Figure 1:**
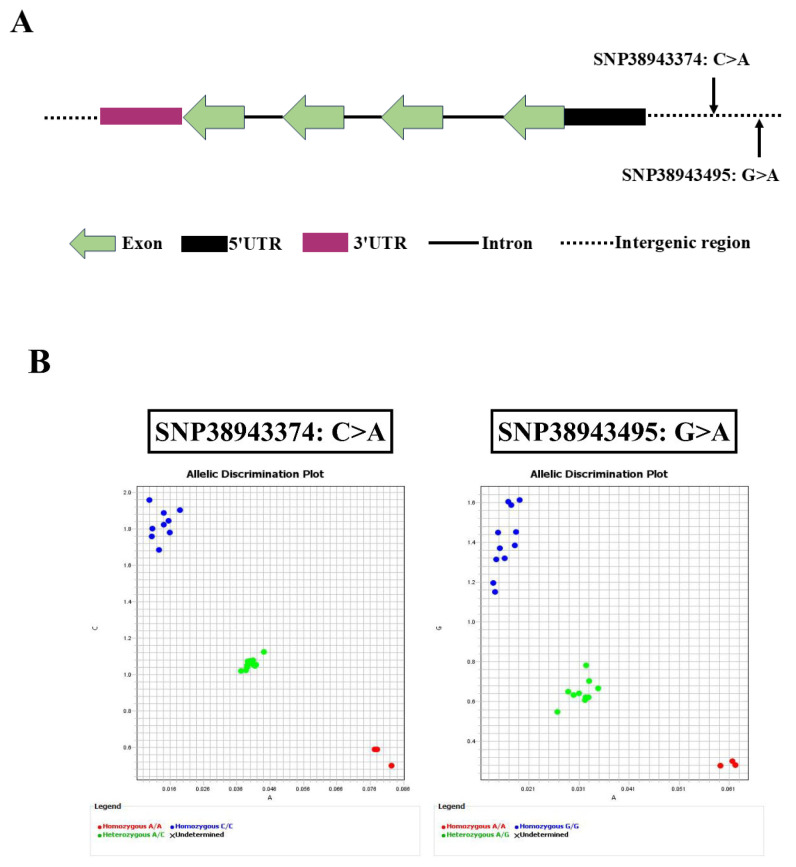
Genetic variations in GADD45b and KASP genotyping. (**A**) The genomic structure and positions of SNPs in GADD45b gene. The SNP loci associated with resistance/susceptibility to LMBV were markerd. (**B**) KASP analysis results for GADD45b genetic variants. Homozygous is indicated by blue and red dots, and heterozygous is shown with green dots.

As shown in Figure 1A, SNP38943374 (C>A) and SNP38943495 (G>A) are located in the intergenic regions of the GADD45b gene. Using the KASP assay, the two markers were found to divide the population into three distinct groups, respectively (Figure 1B). Near the y-axis, the blue clusters indicate homozygous genotypes: the CC group in SNP38943374 and the GG group in SNP38943495 (Figure 1B). Positioned near the x-axis, the red clusters indicate another collection of homozygous genotypes, which in this case were the AA group for SNP38943374 and the AA group for SNP38943495 (Figure 1B). In the central region, the green clusters indicate heterozygous genotypes: AC for SNP38943374 and AG for SNP38943495 (Figure 1B).

### 2.2. Association of GADD45b SNPs with the Resistance/Susceptibility to LMBV

**Figure 2:**
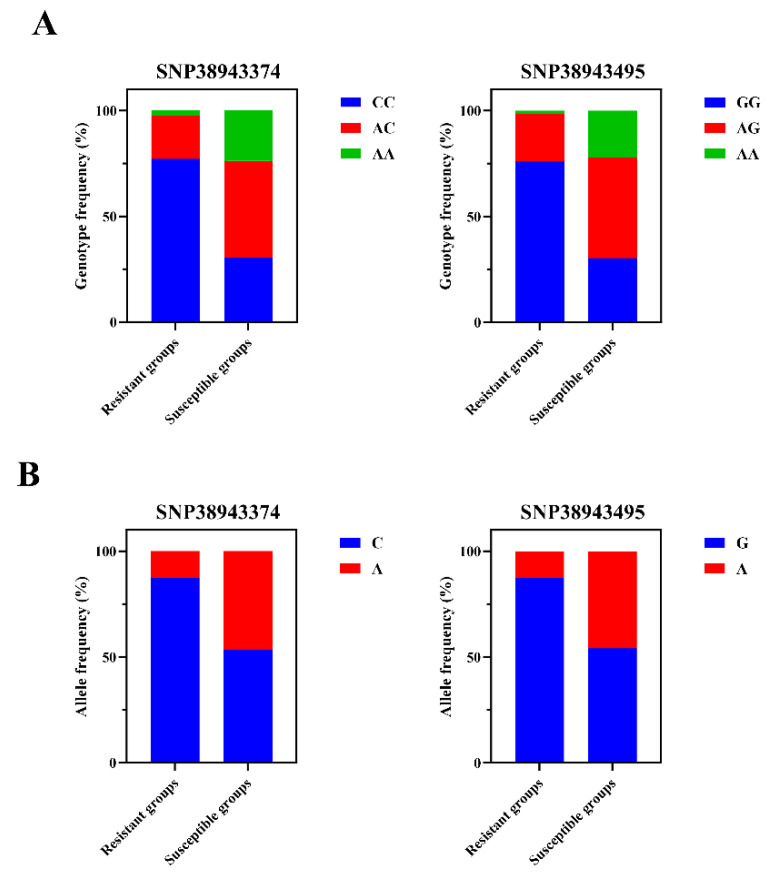
Distribution of genotype and allele frequencies at the two GADD45b SNPs across resistant and susceptible groups. (**A**) Genotype frequency of SNP38943374 C>A and SNP38943495 G>A. (**B**) Allele frequency of SNP38943374 C>A and SNP38943495 G>A.

We genotyped two SNPs in 240 individuals (118 resistant and 122 susceptible) and performed detailed statistical analyses of the genotype and allele frequencies for each SNP (Figure 2). As shown in Table 1 and Table 2, SNP38943374 C>A and SNP38943495 G>A was significantly associated with the resistance/susceptibility to LMBV both in genotype and allele analysis (*p* < 0.01). In resistant groups, the CC, AC and AA genotype frequencies at SNP38943374 C>A were 77.1%, 20.3% and 2.5%, and the GG, AG and AA genotype frequencies at SNP38943495 G>A were 76.3%, 22.0% and 1.7%, respectively, while the corresponding frequency distributions in susceptible groups were 30.3%, 45.9% and 23.8% at SNP38943374 C>A, and 30.3%, 47.5% and 22.1% at SNP38943495 G>A, respectively.

### 2.3. Survival Time Variation Across Genotypes After LMBV Challenge

**Figure 3:**
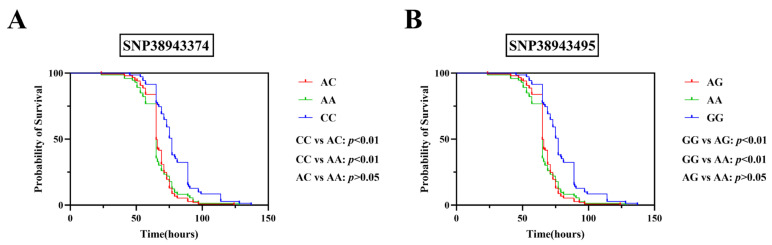
Kaplan–Meier survival analysis of individuals with different genotypes following LMBV challenge. (**A**) In SNP38943374, the number of individuals with the CC genotype is 71, those with the AC genotype is 151, and those with the AA genotype is 74. (**B**) In SNP38943495, the number of individuals with the GG genotype is 71, those with the AG genotype is 151, and those with the AA genotype is 74. *p* value was calculated by the log-rank (Mantel–Cox) test.

Analysis of genotyping results demonstrated significant differences in survival time across genotypes at two loci (SNP38943374 and SNP38943495) of GADD45b following LMBV infection (*p* < 0.01) (Figure 3). The survival time of individuals with the CC genotype at SNP38943374 and the GG genotype at SNP38943495 was significantly longer than that of the other two genotypes individuals (*p* < 0.01). Thus, SNP38943374 and SNP38943495 were significantly associated with resistance to LMBV infection in largemouth bass. There were no significant differences in survival time between individuals with the AC and AA genotypes at SNP38943374 or between those with the AG and AA genotypes at SNP38943495 (*p* > 0.05).

### 2.4. Linkage Disequilibrium Analysis of GADD45b Gene SNPs

**Figure 4:**
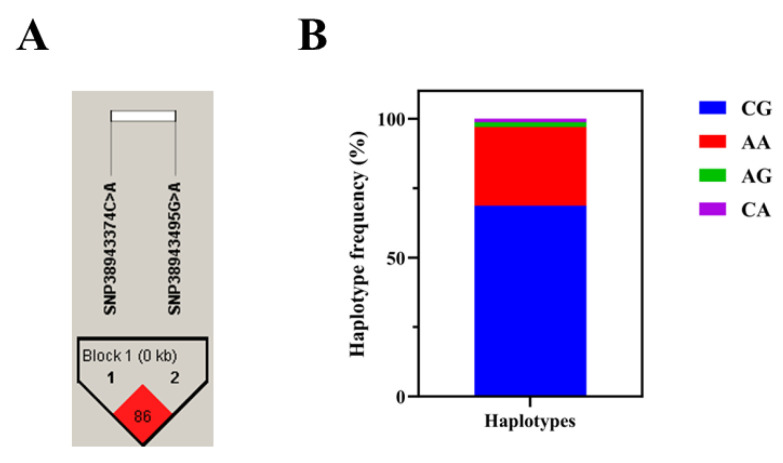
Linkage disequilibrium and haplotype analysis of two SNPs in the GADD45b. (**A**) The values inside the squares indicate the LD values (r^2^) of the two SNPs. The number within each square indicates the r^2^ value multiplied by 100. (**B**) Haplotype frequency distribution of GADD45b gene.

Using Haploview 4.2 software, we analyzed the linkage disequilibrium between two SNPs in the GADD45b gene. The results showed a strong pairwise linkage disequilibrium (r^2^ = 0.86) between loci SNP38943374 C>A and SNP38943495 G>A (Figure 4A). The genotype distributions of these two SNPs are in Hardy–Weinberg equilibrium. Furthermore, haplotype analysis showed that the frequency of the haplotype CG (68.7%) was higher than that of the haplotype AA (28.3%) (Figure 4B).

### 2.5. GADD45b Expression in Different Genotypes After LMBV Infection

**Figure 5:**
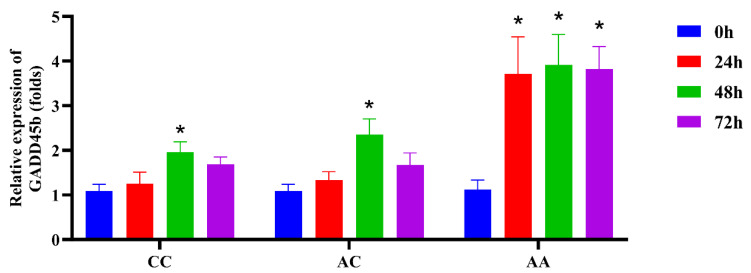
Dynamics expression of GADD45b in different genotypes following LMBV infection. The figure shows GADD45b expression levels post-viral infection across genotypes: CC (equivalent to GG in SNP38943495), AC (equivalent to AG in SNP38943495), and AA (same in both SNPs) of SNP38943374. The data are presented as mean ± SEM (*N* = 8). * *p* < 0.05.

To examine the response of GADD45b gene to LMBV infection, the time-course expression of the GADD45b mRNA following LMBV infection was investigated. After LMBV infection, GADD45b levels increased across all genotype groups from 0 h to 48 h, then decreased from 48 h to 72 h (Figure 5). In the CC and AC genotypes, the expression level of GADD45b exhibited a gradual increase from 0 h to 48 h, peaking at 48 h with a 1.96-fold and 2.35-fold upregulation compared to the control group, respectively (*p* < 0.05). Differently, the AA group maintained consistently high GADD45b expression levels at 24 h, 48 h, and 72 h after viral infection, reaching 3.71-, 3.91-, and 3.82-fold of the control group, respectively (*p* < 0.05). Overall, the AA group exhibited higher GADD45b expression levels following viral infection compared to the CC and AC groups.

### 2.6. Immune Gene Expression Profiles Across Genotypes Following LMBV Infection

**Figure 6:**
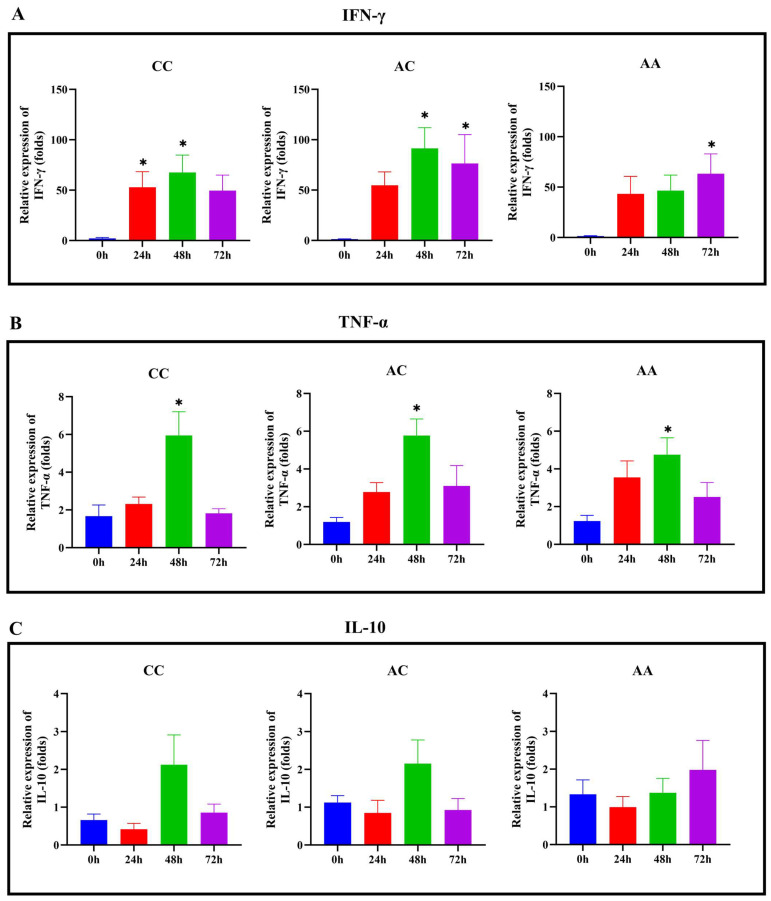
Dynamics expression of IFN-γ, TNF-α and IL-10 in different genotypes after LMBV infection. qRT-PCR was performed to analyze the expression levels of IFN-γ (**A**), TNF-α (**B**) and IL-10 (**C**). CC group: individuals with the genotype of SNP38943374, as well as individuals with the GG genotype of SNP38943495. AC group: individuals with the genotype of SNP38943374, as well as individuals with the AG genotype of SNP38943495. AA group: individuals with the genotype of SNP38943374, as well as individuals with the AA genotype of SNP38943495. The data are presented as mean ± SEM (*N* = 8). * *p* < 0.05.

We further analyzed the expression changes of immune-related genes (including IFN-γ, TNF-α, and IL-10) in different genotypes following LMBV infection. As shown in Figure 6A, IFN-γ expression in the CC and AC groups peaked at 48 h post-viral infection, reaching levels 67.57-fold and 91.49-fold higher than the control group, respectively (*p* < 0.05). In contrast, the AA group exhibited peak IFN-γ expression at 72 h, with a 63.37-fold increase compared to the control group (*p* < 0.05). As shown in Figure 6B, TNF-α expression reached peak levels at 48 h, showing significant increases to 5.95-fold, 5.77-fold, and 4.75-fold of the control group in the CC, AC, and AA groups, respectively (*p* < 0.05). As demonstrated in Figure 6C, IL-10 expression decreased from 0 h to 24 h post-viral infection in all three groups. Subsequently, IL-10 levels in the CC and AC groups increased between 24 h and 48 h, followed by a decline from 48 h to 72 h. In contrast, the AA group exhibited a sustained increase in IL-10 expression from 24 h to 72 h (*p* > 0.05).

## 3. Discussion

GADD45b is a pivotal cellular signaling regulator that has been documented to participate in multiple biological processes, including autophagy, apoptosis, and immune regulation [20,21,22,25]. The intensive culture of largemouth bass has been severely limited by recurrent outbreaks of LMBV. Selective breeding, especially genomic selection based on genetic variants, is considered an effective strategy to enhance the disease resistance of fish [31,32]. Currently, several new aquatic disease-resistant varieties have been successfully developed using genomic selection technology with molecular markers, including a red seabream strain resistant to red sea bream iridoviral disease (RSIVD) [33], a strain of Japanese flounder resistant to lymphocystis disease [34], and a strain of *Cynoglossus semilaevis* resistant to *Vibrio harveyi* [35]. In light of the critical function of GADD45b in viral infection and host immune regulation, it is valuable to explore the relationship between GADD45b gene polymorphisms and disease resistance in largemouth bass.

SNP markers are widely used as genetic markers in molecular marker-assisted selection in aquaculture species [36]. In recent years, the polymorphism of immune-related genes in an increasing number of aquaculture species has been demonstrated to be associated with resistance/susceptibility to various diseases, such as IL-6 in mandarin fish [11], TLR8b in grass carp [13], MHC IIα in Japanese flounder and golden pompano [12,37]. The discovery of these immune-related gene polymorphisms provides valuable molecular marker resources for disease-resistant breeding in fish. Nevertheless, research focusing on the characterization of GADD45b polymorphism in fish is limited.

To our knowledge, this is the first study to identify an association between GADD45b SNPs and disease susceptibility in fish. Currently, the main methods for SNP genotyping based on PCR technology include: amplification refractory mutation system PCR (ARMS-PCR) [38], high-resolution melting analysis (HRMA) [39], TaqMan probe method [40] and kompetitive allele specific PCR (KASP) [41]. Considering the advantages of the KASP method, including low cost, high accuracy and operational simplicity, we employed the KASP method for the first time to genotype two SNP loci in the GADD45b. And the statistical analyses indicated that the genotypes and alleles of SNP38943374 C>A and SNP38943495 G>A were significantly associated with largemouth bass resistance to LMBV (*p* < 0.05) (Table 1). In addition, the CC genotype of SNP38943374 C>A and the GG genotype of SNP38943495 G>A are more prevalent in resistant groups. Survival time is an important indicator used to analyze the association between genetic variants and traits. For example, through the analysis of survival time among individuals with different genotypes, the researchers validated the SNP (T/C) locus associated with ammonia nitrogen tolerance in Nile tilapia, as well as the SNP24194184 and SNP24101852 loci linked to low oxygen tolerance in golden pompano [4,42]. In this work, individuals with the CC genotype of SNP38943374 C>A and the GG genotype of SNP38943495 G>A had significantly longer survival times after viral infection compared to that of the other two genotypes individuals. Additionally, we observed a strong linkage disequilibrium between these two SNP loci, suggesting they may share common genetic associations and implying their equally important roles in resisting viral infections. And the haplotype analysis revealed that the CG and AA haplotypes are associated with LMBV resistance/susceptibility. These results suggested that the C allele of SNP38943374 C>A and the G allele of SNP38943495 G>A could be the genetic protective factors against LMBV infection.

Among the two SNP loci in the GADD45b, SNP38943374 (C>A) and SNP38943495 (G>A) are located in intergenic regions. For an extended period, intergenic genomic sequences have been conventionally characterized as “junk” DNA segments. However, researchers have discovered that intergenic regions containing SNP loci displayed transcriptional activity [43,44]. SNP loci located in intergenic regions may contribute to major depressive disorder susceptibility by modulating long non-coding RNA expression [45]. On the other hand, intergenic SNP loci exhibited strong linkage disequilibrium could serve as genetic markers for disease association studies [30], although they may not have a direct functional role. Previous studies have reported that intergenic regions upstream of GADD45b have been reported to harbor conserved regulatory elements, such as enhancers and transcription factor binding sites (e.g., p53, NF-κB) [16]. These regions may modulate stress-responsive transcriptional activity by facilitating chromatin remodeling or serving as docking sites for co-regulators. In this work, the disease-associated SNP38943374 and SNP38943495 in intergenic regions upstream of GADD45b may play functional roles in transcriptional activity regulation or serve as genetic markers for monitoring LMBV risk.

In the present work, GADD45b expression is upregulated in response to LMBV infection in different genotype individuals, aligning with the induction of GADD45b observed in HIV-1 and ALV-J viral infections [18,19]. Remarkably, compared to individuals with the other two genotypes, those with the AA genotype of SNP38943374 C>A or the AA genotype of SNP38943495 G>A showed higher expression levels of GADD45b following viral infection. These results indicated that individuals with the AA genotype show a more pronounced response to viral infection. Virus-induced differential expression of GADD45b among different genotype populations may implicate its involvement in the pathogenic mechanisms underlying LMBV. However, how GADD45b contributes to LMBV development remains elusive.

The antiviral functions of IFN-γ and TNF-α as pro-inflammatory cytokines have been investigated in various fish species [46,47,48,49]. In this study, we found that individuals with the AA genotype of SNP38943374 and SNP38943495 displayed lower overall expression levels of IFN-γ and TNF-α during the early phase of viral infection (from 0 h to 48 h) compared to those with the other two genotypes. This may be the reason why AA genotype individuals were susceptible to LMBV, as they were unable to clear the pathogen in the early stages of viral infection. Studies have demonstrated that GADD45b inhibits apoptosis mediated by TNF-α [50]. Based on the results showing that GADD45b is highly expressed after viral infection in combination with the AA genotype of SNP38943374 and SNP38943495, we speculate that upregulated GADD45b may play a regulatory role in viral infection by modulating TNF-α-mediated apoptosis. LMBV-induced GADD45b expression may suppress TNF-α-mediated apoptosis, thereby prolonging host cell survival and providing an extended ‘factory’ period for viral replication. Conversely, this delayed apoptosis allows more time for the virus to complete its replication, assembly, and release processes, ultimately leading to increased viral titers and promoting the spread of viral infection. Additionally, IL-10, as an anti-inflammatory cytokine, has been demonstrated to downregulate pro-inflammatory factors in fish, thereby inhibiting inflammatory responses and mitigating inflammation-induced tissue damage [51,52]. In our study, AC and CC genotype individuals showed a rapid increase in IL-10 expression during the early stages of viral infection, suggesting that these genotype individuals may be able to rapidly initiate IL-10 expression in response to the inflammatory response triggered by infection. In contrast, AA genotype individuals exhibited a sustained increase in IL-10 expression during the late stages of viral infection. The sustained increase in IL-10 expression may keep the host in a state of strong immune suppression during the later stages of infection, which may affect the efficiency of pathogen clearance and even lead to chronic infection or persistent infection.

Therefore, future studies on GADD45b should focus on clarifying its antiviral mechanisms, particularly how it modulates cellular signaling to induce antiviral factors and regulate host immune responses against LMBV. Further studies are also needed to investigate how intergenic SNPs regulate gene expression, particularly through experimental validation—such as dual-luciferase reporter assays or ATAC-seq—to establish causal relationships. On the other hand, given the genetic diversity of GADD45b and its association with LMBV resistance/susceptibility, selective breeding of individuals carrying resistance-associated genotypes could facilitate the development of a largemouth bass strain with enhanced resistance to LMBV, thereby mitigating the impact of viral disease in aquaculture. Additionally, it would be valuable to evaluate potential trade-offs in growth rate and reproductive performance in LMBV-resistant lines to ensure comprehensive breeding success.

## 4. Materials and Methods

### 4.1. Ethics Statement

All animal experiments were conducted in accordance with the ethical guidelines approved by the Pearl River Fisheries Research Institute’s Animal Ethics Committee (Approval No. LAEC-PRFRI-2024-02-01).

### 4.2. Experimental Animals and Virus

Juvenile largemouth bass (with a weight of about 5.33 g and a body length of about 6.10 cm) used in the study were collected from Foshan City, Guangdong Province, China. Prior to the experiment, all fish were maintained in a circulating aquaculture tank at 28 ± 0.5 °C with >6 mg/L dissolved oxygen, and fed commercial pellets (Yuequn, Jieyang, Guangdong, China) twice daily (2% body weight) for 14 days. Chinese perch brain (CPB) cell line sensitive to LMBV (strain 2007064) was established and maintained in our laboratory, cultured in L-15 medium (Gibco, Waltham, MA, USA) supplemented with 10% FBS at 28 °C [53]. LMBV was isolated and stored at −80 °C in our laboratory [26]. The virus was propagated in CPB cells at a temperature of 28 °C, and its titer was measured using the 50% tissue culture infectious dose (TCID_50_) assay.

### 4.3. LMBV Infection and Sample Collection

To verify the association between the polymorphism of the GADD45b gene in largemouth bass and LMBV resistance/susceptibility, we selected 240 fish infected with the virus as the study subjects. Largemouth bass that survived for more than 14 days after viral challenge were considered resistant individuals, while those that died within 14 days along with typical LMBV symptoms (e.g., body darkening, significant spleen/liver enlargement) were deemed susceptible [30]. For fish that succumbed to infection, caudal-fin tissue samples were collected immediately upon death and subjected to gross pathological examination, while survivors were sampled 14 days post-infection. In order to study dynamic gene expression profiles of different genotypes, 420 largemouth bass were used for virus infection experiments. For viral infection, the fish were anesthetized with MS-222 (Sigma, Burbank, CA, USA) before intraperitoneal injection. 400 individuals in the experimental group were injected intraperitoneally with 100 µL LMBV (3.98 × 10^5^ TCID_50_/mL), suspended in PBS. 20 individuals in the control group were injected with PBS. Approximate 0.1 g liver tissue and caudal fin tissue were collected immediately at 0, 24, 48, and 72 h post-LMBV infection. For the genotype-survival correlation analysis, 296 fish were intraperitoneally injected with 100 µL LMBV (10^7^ TCID_50_/mL) and the survival time of each fish after virus infection was recorded. In the genotype-survival correlation analysis experiment, all individuals exhibited 100% mortality under high-titer viral challenge. Approximate 0.1 g caudal fin tissue were collected for genotyping.

### 4.4. DNA/RNA Extraction and qRT-PCR Analysis

According to the manufacturer’s instructions, the genomic DNA was isolated by using the magnetic bead-based genomic DNA extraction kit (BayBio, Guangzhou, China) and the RNA was isolated by using the Animal Total RNA Isolation Kit (Foregene, Chengdu, China). The concentration and quality of the DNA/RNA were assessed using the microplate reader (BioTek, Shoreline, WA, USA). RNA quality was assessed via agarose gel electrophoresis. 1 μg of total RNA from each sample was used for cDNA synthesis using TransScript^®^ Uni All-in-One First-Strand cDNA Synthesis SuperMix for qPCR kit (TransGen Biotech, Beijing, China). qPCR was performed under standard conditions (95 °C for 1 min, followed by 40 cycles of 95 °C for 5 s, 60 °C for 30 s) using SYBR Green (Accurate, Changsha, China). The Applied Biosystems^®^ QuantStudio 6 platform (Thermo Fisher Scientific, Waltham, MA, USA) was employed for qRT-PCR analysis. 18S rRNA were utilized as internal reference genes for normalization and the expression levels were calculated using the 2^−ΔΔCt^ method. Primer sequences are provided in Table 3. The primer validation is shown in Figure S1.

### 4.5. Genotyping SNPs in the GADD45b Gene with KASP

Kompetitive allele specific PCR (KASP) method is used to genotype SNPs in GADD45b gene. As shown in Table 3, KASP primers were designed for the two SNP loci (SNP38943374 C>A and SNP38943495 G>A) in GADD45b. The detailed information of these two SNP loci is presented in Table 4. For every SNP locus, the KASP primers included three sequences: a pair of allele-specific forward primers, one labeled with FAM and the other with HEX, along with a single common reverse primer (Table 3). The genotyping analysis was conducted using the KASP assay on the Applied Biosystems^®^ QuantStudio 6 platform (Thermo Fisher Scientific, USA).

### 4.6. Association Analysis Between GADD45b SNPs and Resistance/Susceptibility to LMBV

We analyzed the genotyping results to calculate the genotype and allele frequencies for each SNP loci. The chi-square test (χ^2^ test) was used to evaluate genotype polymorphism and explore the association between GADD45b and LMBV resistance/susceptibility in largemouth bass. Statistical analysis was conducted using the chi-square test in SPSS 27.0.1 (Statistical Program for Social Science, SPSS Inc., Chicago, IL, USA). Additionally, we conducted linkage disequilibrium (LD) analysis and Hardy–Weinberg equilibrium (HWE) of SNP loci using HaploView 4.2 software. The extent of linkage disequilibrium between loci is determined by r^2^. When r^2^ > 0.33, it indicates a strong linkage relationship between loci [6].

### 4.7. Statistical Analysis

GraphPad Prism 10.1 (GraphPad Software, Inc., San Diego, CA, USA) was employed for statistical analysis of the data. Gene expression data are represented as mean ± standard error of the mean (SEM). We applied a one-way ANOVA to test for significant differences between the groups. * *p* < 0.05, ** *p* < 0.01.

## 5. Conclusions

In summary, we investigated the association between two SNP loci (SNP38943374 C>A and SNP38943495 G>A) in the GADD45b gene and the resistance or susceptibility of largemouth bass to LMBV. Individuals with the CC genotype of SNP38943374 and the GG genotype of SNP38943495 were more prevalent in resistant groups and have advantages in survival time after LMBV infection. These findings provide candidate markers for selective breeding of resistant largemouth bass against LMBV.

## Figures and Tables

**Table 1:** The genotype frequency distribution of GADD45b gene polymorphisms in resistant and susceptible groups.

Locus	Genotype	Resistant Groups No. (%)	Susceptible Groups No. (%)	χ^2^ (*p*)
SNP38943374 C>A	CC	91 (0.771)	37 (0.303)	56.655 (*p* < 0.01)
	AC	24 (0.203)	56 (0.459)	
	AA	3 (0.025)	29 (0.238)	
SNP38943495 G>A	GG	90 (0.763)	37 (0.303)	55.809 (*p* < 0.01)
	AG	26 (0.220)	58 (0.475)	
	AA	2 (0.017)	27 (0.221)	

**Table 2:** The allele frequency distribution of GADD45b gene polymorphisms in resistant and susceptible groups.

Locus	Allele	Resistant Groups No. (%)	Susceptible Groups No. (%)	χ^2^ (*p*)
SNP38943374 C>A	C	206 (0.873)	130 (0.533)	66.075 (*p* < 0.01)
	A	30 (0.127)	114 (0.467)	
SNP38943495 G>A	G	206 (0.873)	132 (0.541)	63.438 (*p* < 0.01)
	A	30 (0.127)	112 (0.459)	

**Table 3:** The primers of this study.

Primer	Sequence (5′-3′)
SNP38943374-F1	GAAGGTGACCAAGTTCATGCTGATGATGAGGTGCCAGGGTGGAGGC
SNP38943374-F2	GAAGGTCGGAGTCAACGGATTGATGATGAGGTGCCAGGGTGGAGGA
SNP38943374-R	GAAGGCTGTAACTGCTGAGCGGAAGTGTT
SNP38943495-F1	GAAGGTGACCAAGTTCATGCTACATTAAAATATCACATGACATAAG
SNP38943495-F2	GAAGGTCGGAGTCAACGGATTACATTAAAATATCACATGACATAAA
SNP38943495-R	TTTCAGCTCTGGTTTGGTTGTAAGAGTAT
GADD45b-RT-F	CTTTCTGCTGCGACAACGAC
GADD45b-RT-R	GAGGGTTCGTGACCAGGATG
IFN-γ-RT-F	TGCAGGCTCTCAAACACATC
IFN-γ-RT-R	TGTTTTCGGTCAGTGTGCTC
TNF-α-RT-F	ATCTGCTGTGAATGCCGTGA
TNF-α-RT-R	CGTCAGCCTGGATAGACGAC
IL-10-RT-F	CTAGACCAGAGCGTCGAGGA
IL-10-RT-R	CCAAGGCTGTTGGCAGAATC
18s-RT-F	TGACGGAAGGGCACCACCAG
18s-RT-R	GCACCACCACCCACAGAATCG

**Table 4:** The information of two SNP loci in the GADD45b gene.

SNP	Chromosome	Locus	Ref	Alt	Region
SNP38943374 C>A	NW_024044237.1	38943374	C	A	Intergenic
SNP38943495 G>A		38943495	G	A	Intergenic

## Data Availability

The original contributions presented in this study are included in the article/Supplementary Materials. Further inquiries can be directed to the corresponding author.

## Supplementary Materials

The following supporting information can be downloaded at: https://www.mdpi.com/article/10.3390/ijms26199281/s1.
