# Supplementary material for: Intergenic Variants Upstream of GADD45b Affect Survival of Micropterus salmoides Following LMBV Exposure
Source: Int J Mol Sci. 2025 Sep 23;26(19):9281. doi: 10.3390/ijms26199281 (PMC12525184; doi:10.3390/ijms26199281)
Supplement: Supplementary file 1 [file ijms-26-09281-s001.zip › ijms-3790921-supplementary.pdf]

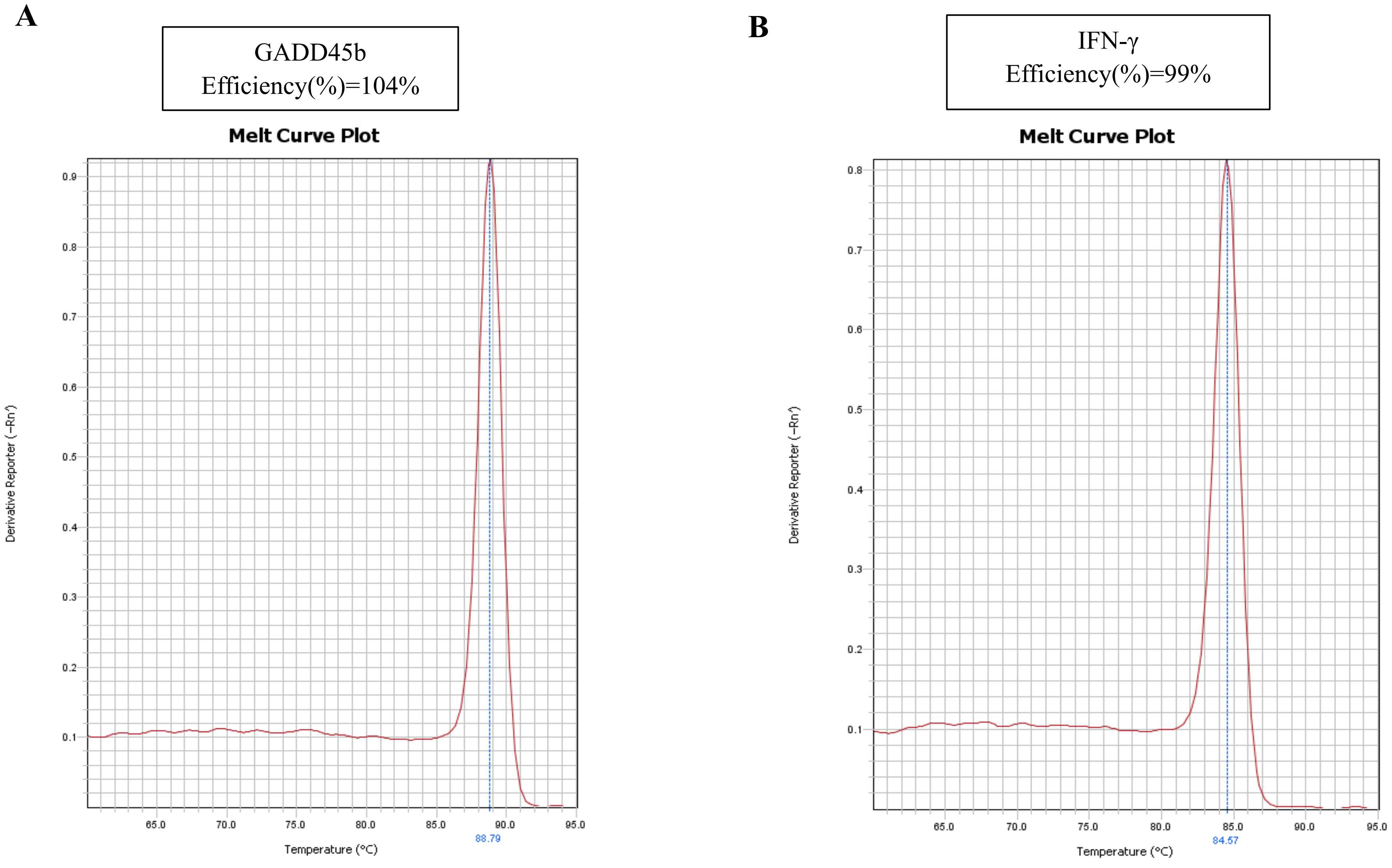
A
GADD45b
Efficiency(%)=104%
Melt Curve Plot
Derivative Reporter (−Rn')
0.9
0.8
0.7
0.6
0.5
0.4
0.3
0.2
0.1
65.0
70.0
75.0
80.0
85.0
90.0
95.0
88.79
Temperature (°C)
B
IFN-γ
Efficiency(%)=99%
Melt Curve Plot
Derivative Reporter (−Rn')
0.8
0.7
0.6
0.5
0.4
0.3
0.2
0.1
65.0
70.0
75.0
80.0
85.0
90.0
95.0
84.57
Temperature (°C)

C

TNF-α
Efficiency(%)=101%

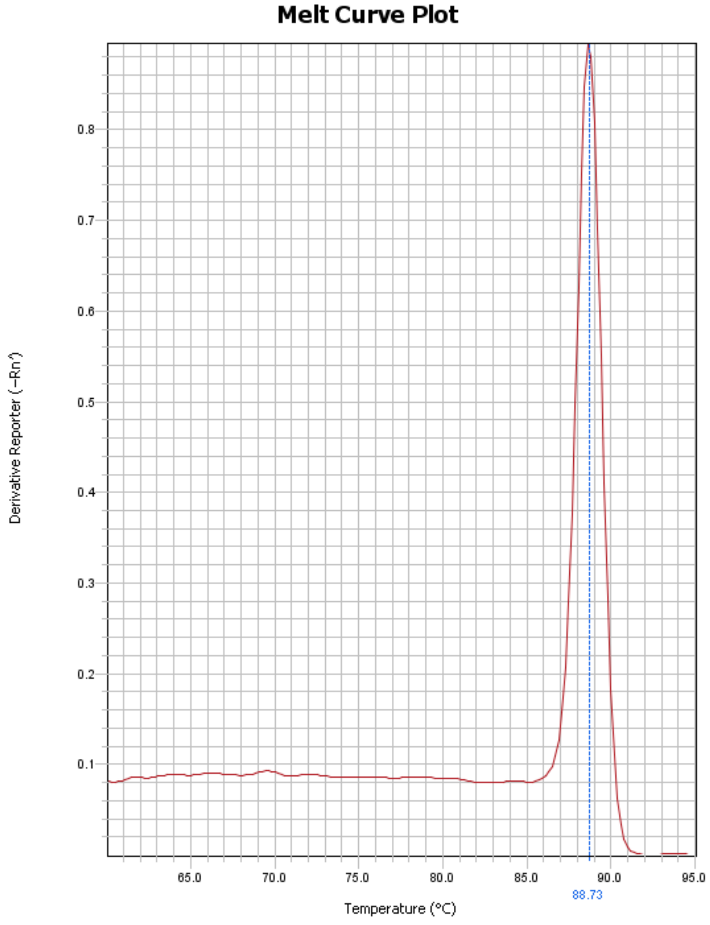

D

IL-10
Efficiency(%)=99%

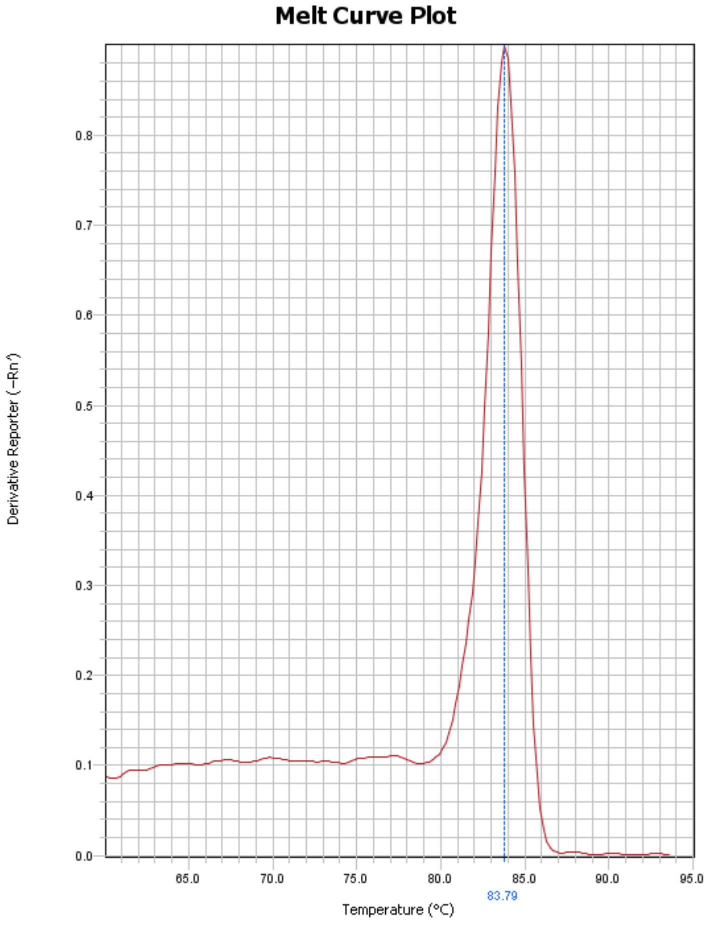

E

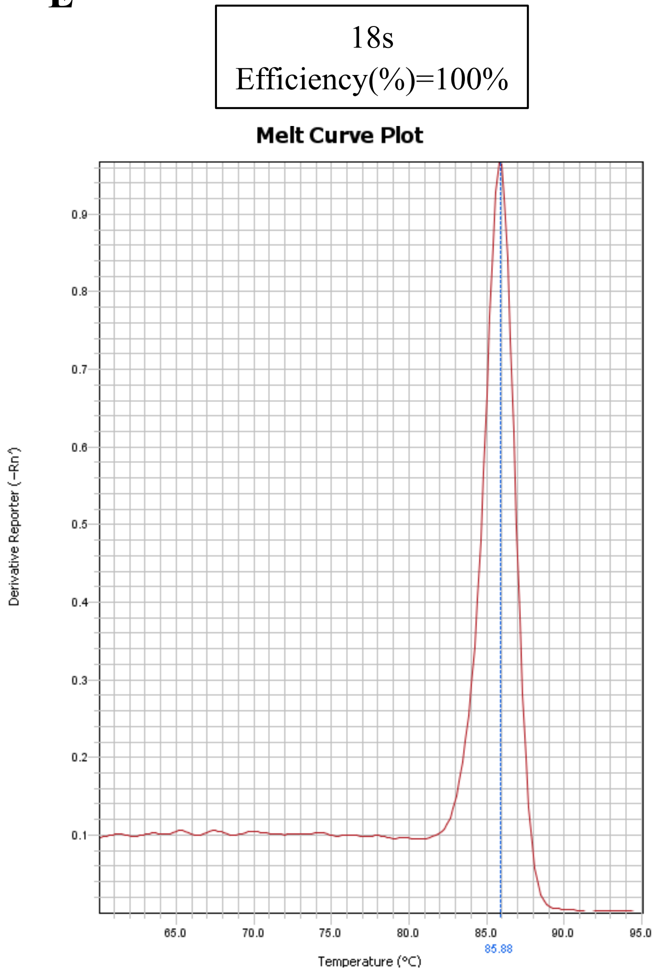

Figure S1. The primer efficiency and melting curve analysis of GADD45b (A), IFN-γ (B), TNF-α (C), IL-10 (D) and 18s (E).
